# Characteristics of Gut Microbiota in Female Patients with Diabetic Microvascular Complications

**DOI:** 10.1155/2022/2980228

**Published:** 2022-10-27

**Authors:** Shan Gao, Li-hua Zhao, Xue Tian, Mo-wei Kong, Jian-qiu He, Xiao-chun Ge, Xiao-yan Liu, Zeng-bin Feng, Yu Gao

**Affiliations:** ^1^Department of Endocrinology, Affiliated Hospital of Chengde Medical University, Chengde, Hebei, China; ^2^Department of Cardiac Surgery, Affiliated Hospital of Chengde Medical University, Chengde, Hebei, China

## Abstract

**Objective:**

To explore the characteristics and analyze the gut microbiota in female patients with diabetic microvascular complications (DMC).

**Methods:**

Thirty-seven female patients with type 2 diabetes mellitus (T2DM) were included in the study. These patients were divided into DM group with microvascular complications (T2DM-MC, *n* = 17) and no microvascular complications group (T2DM-0, *n* = 20). Patients in the microvascular group presented with the involvement of at least one of the following: kidney, retinal, or peripheral nerves. Using real-time fluorescence quantitative polymerase chain reaction, fecal samples from the two groups were tested for *Bacteroides*, *Prevotella*, *Bifidobacterium* spp, *Lactobacillus*, *Faecalibacterium prausnitzii, Enterococcus* spp, *Eubacterium rectale, Veillonellaceae*, *Clostridium leptum*, and *Roseburia inulinivorans*. Levels of fasting and 2 h postprandial blood glucose, glycosylated hemoglobin (HbA1c), lipids, and creatinine were determined to explore the correlation between gut microbiota and blood sugar. Mann–Whitney *U* test was used to analyze the differences between the two groups. Spearman correlation analysis was used to determine the correlation between gut microbiota and blood glucose. Multifactor logistic regression was used to analyze the risk factors for DMC.

**Results:**

The HbA1c levels in the T2DM-MC group were higher than those in the T2DM-0 group. The abundances of *Bacteroides* and *Enterococcus* spp in the T2DM-MC group were higher than that in the T2DM-0 group. The abundances of *Bacteroides* and *Enterococcus* spp in the T2DM-MC group were lower than that in the T2DM-0 group. Spearman's correlation analysis showed that *Bacteroides*, *Prevotella*, *Lactobacillus*, *C. leptum*, and *R. inulinivorans* were related to the levels of HbA1c or blood glucose (*p* < 0.05). Logistic regression analysis showed that after adjusting for confounding factors such as age, body mass index, family history, HbA1c, hypertension, dyslipidemia, and creatinine, *Bacteroides* remained an independent risk factor in female patients with DMC.

**Conclusion:**

Gut microbiota is related to blood glucose levels. Female patients with DMC experience gut microbiota disorders. The abundances of *Bacteroides*are related to DMC, and the abundances of intestinal flora may affect the blood sugar levels of the body.

## 1. Introduction

Diabetes mellitus (DM), if poorly controlled for more than 10 years from the onset, is often prone to diabetic microvascular complications (DMC), involving the retina, kidney, and peripheral nerves. In recent years, the ongoing research regarding the involvement of gut microbiota in the pathogenesis of DMC has revealed that the occurrence of DMC may be exacerbated in both deletion and excessive proliferation of different intestinal bacteria.

More recently, the products of bacterial metabolism have been shown to influence the occurrence and progression of chronic kidney disease [1]. In a clinical trial conducted in patients with chronic kidney disease, urea nitrogen in the blood and uric acid concentration decreased after administering a mixture of *Lactobacillus acidophilus*, *Streptococcus thermophilus*, and *Bifidobacterium longum* for six months [2]. Likewise, several studies have reported an association between the imbalance of the gut microbiota and diabetic retinopathy [3, 4]. Recent research on oral probiotic treatment in patients with diabetic retinopathy has shown a decrease in intraocular pressure (IOP) and relief in symptoms [5]. Additionally, changes in the diversity of the gut microbiota and the increased presence of pathogens have been also linked to diabetic neuropathy [6].

Currently, studies on female type 2 diabetes mellitus (T2DM) patients have also shown that intestinal flora imbalance is a potential problem [7, 8]. Most studies have focused on the characteristics of gut microbiota in pregnant women with gestational diabetes mellitus [9–11]. Studies on the relationship between intestinal flora and female patients with DMC are limited. This study aimed to explore the characteristics and correlation analysis of differential bacterial genera, with clinical diagnostic value, in female patients with DMC.

## 2. Materials and Methods

### 2.1. Subjects

In a cohort study, only female patients with T2DM were enrolled and were retrospectively analyzed in the Endocrinology Department of Chengde Medical University, between September 2019 and October 2020. Patients were divided into diabetic microvascular complications group (T2DM-MC, *n* = 17) and no diabetic microvascular complications group (T2DM-0, *n* = 20). All patients with incident T2DM were defined using diagnostic codes from the 2017 Edition of China's Guidelines for the Prevention and Treatment of type 2 diabetes [12]. Exclusion criteria were as follows: with other types of diabetes; renal, retinal, or peripheral neuropathy due to other causes, with a history of gastrointestinal diseases or surgery, and who had taken probiotics, prebiotics, and other such products in the past four weeks; treatment with steroids or immunomodulatory drugs; thyroid disease; severe cardiovascular, hepatic, and renal insufficiency; tumors. The study was conducted in accordance with the principles of the Declaration of Helsinki and was approved by the hospital ethics committee. All the participants provided written informed consent prior to any study-related procedures.

### 2.2. Definitions

**Figure 1:**
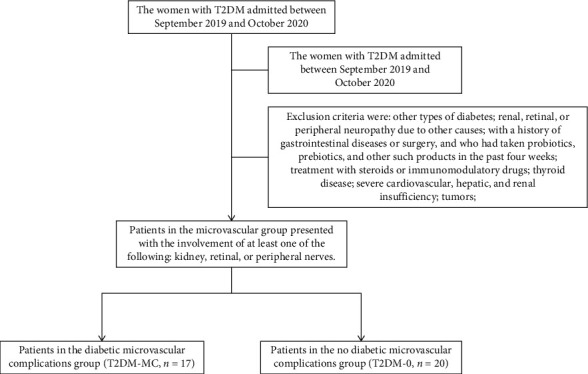
Flow graph illustrating study process.

Patients in the microvascular group presented with the involvement of at least one of the following: kidney, retinal, or peripheral nerves. The diagnostic criteria for diabetes retinopathy (DR) were as follows [13]: combined with clinical manifestations, fundus examination, mydriasis, and fundus angiography to confirm fundus lesions. The diagnostic criteria for diabetic nephropathy (DN) were as follows [14]: (i) urine albumin-creatinine ratio ≥ 30 mg/g for more than three months, or (ii) urinary albumin to creatinine ratio of 30–300 mg/g and concurrent DR. The diagnostic criteria for diabetic peripheral neuropathy (DPN) were as follows [12]: (i) definite diabetic history, (ii) neuropathy after the diagnosis of diabetes, (iii) clinical symptoms (such as pain, numbness, and paresthesia), one of the five listed abnormalities (ankle reflex, acupuncture pain, vibration, pressure, and temperature), and for those without clinical symptoms, two abnormalities in the five listed tests (Figure 1).

## 3. Methods

### 3.1. HbA1c and Biochemical Measurements

General information was collected from all the participants. Blood samples were collected from the antecubital vein at 6 am the next morning after admission, and patient samples were analyzed for glycated hemoglobin (HbA1c), using BIO-RAD D110 (Hercules, CA, USA). The blood samples were also analyzed for the levels of glucose, lipids, and creatinine, using Beckman Coulter AU5800 (Beckman Coulter, Inc., Brea, CA, USA), a fully automatic biochemistry analyzer.

### 3.2. Fecal DNA Extraction and Real-Time Fluorescence Quantitative PCR

Fecal DNA was extracted after pretreatment using an automatic nucleic acid extractor (NP968, Tianlong Technology, Shanxi, China) with the help of a Nucleic Acid Extraction Kit (Tianlong Technology, Shanxi, China). All protocols were performed according to the manufacturer's instructions. Custom-designed primers were used for 10 intestinal bacterial genera according to the bacterial 16S RNA V4 sequences (Baoto Biotechnology, Shanghai, China).  Table 1 shows the sequences for all primer sets used in this study. ABI7500 quantitative polymerase chain reaction (qPCR) instrument (Thermo Fisher Scientific, Rocklin, CA, USA) was used, following the manufacturer's instructions for the real-time fluorescent qPCR amplification experiments to verify the sequencing results; the bacterial content was calculated using the 2 − ΔΔ*Ct* method.

### 3.3. Statistical Analyses

Statistical analyses were performed using the SPSS software (ver.25.0; SPSS Inc.). Data are expressed as mean ± standard deviation for continuous variables with normal distribution, median and interquartile spacing (M (Q1, Q3)) for those with skewed distribution, and frequencies (proportions) for categorical variables. Continuous variables were compared with the independent sample *t*-test or the Mann–Whitney *U* test. Categorical variables were compared using the chi-squared test. Spearman correlation analysis was used to determine the correlation between intestinal flora content, HbA1c levels, and blood sugar levels. Logistic regression analyzed the disease risk factors in female patients with DMC. A *p* value <0.05 was considered to indicate statistical significance in all the tests.

## 4. Results

### 4.1. Comparison of Patient Clinical Characteristics

A total of 20 T2DM patients and 17 T2DM with DMC patients were included in this study. Demographic, clinical, and laboratory parameters and statistical results for comparisons of the groups are shown in Table 2. HbA1c levels were significantly higher in the T2DM-MC group than in the T2DM-0 group (*p* < 0.05). Age, body mass index (BMI), family history, fasting blood glucose (FBG), 2 h plasma glucose (2hPG), hypertension, dyslipidemia, DMC, and the use of antidiabetic agents were not significantly different between the two groups (*p* > 0.05, Table 2).

### 4.2. Comparison of Intestinal Bacterial Genus Content between the Two Groups

The abundances of *Bacteroides* and *Enterococcus* were higher in the females in the T2DM-MC group than those in the T2DM-0 group (*p* < 0.05), and the content of *Lactobacillus* was lower in the females in the T2DM-MC group than those in the T2DM-0 group (*p* < 0.05). There was no significant difference in the abundances of other intestinal bacteria between the two groups (*p* > 0.05) (Table 3).

### 4.3. Correlations between Gut Bacterial Genera and Blood Glucose

**Figure 2:**
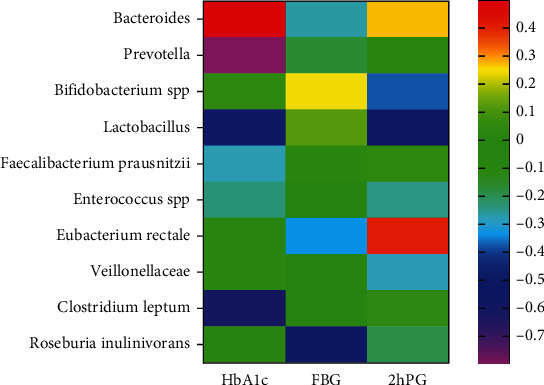
Correlation analysis of gut bacterial genera with blood glucose levels and HbA1c. HbA1c: glycosylated hemoglobin; FBG: fasting blood glucose; 2hPG: 2-hour plasma glucose.

Spearman correlation analysis showed that the presence of *Bacteroides* in females was positively associated with the HbA1c levels (*p* < 0.05, *r* = 0.496); *Prevotella* was negatively associated with HbA1c levels (*p* < 0.05, *r* = −0.79); *Lactobacillus* was negatively associated with HbA1c levels (*p* < 0.05, *r* = −0.489); *Lactobacillus* was negatively associated with 2hPG levels (*p* < 0.05, *r* = −0.515); *Clostridium leptum* was negatively associated with HbA1c levels (*P* < 0.05, *r* = −0.609), and *Roseburia inulinivorans* was negatively associated with FBG levels (*p* < 0.05, *r* = −0.564) (Figure 2).

### 4.4. Risk Factors in Female Patients with DMC

Based on whether DMC was considered as the dependent variable, *Bacteroides* was used as the independent variable, and logistic regression analysis was performed. The logistic regression model showed that the risk of DMC increased with an increase in the abundance of *Bacteroides* genus [odds ratio (OR) = 1.045, 95% confidence interval (CI) = 1.002–1.090). After adjusting for confounding factors, including age, BMI, family history, HbA1c levels, hypertension, dyslipidemia, and creatinine levels, *Bacteroides* remained an independent risk factor for DMC (OR = 1.130, 95% CI = 1.007–1.269, Table 4).

## 5. Discussion

Studies have shown that intestinal microbiota may play a role in the development of complications in type 2 diabetic individuals [15]. The dynamic balance of the intestinal microecological environment is destroyed with a change in the proportion of probiotics in the body. This induces an inflammatory response and the occurrence of DMC, which is closely related to an increase in inflammatory factors such as interleukin (IL)-1 beta, monocyte chemoattractant protein-1, cell adhesion molecule-1, IL-8, and C-reactive protein [16, 17]. In previous study, *Lactobacillus* had been used to lessen the risk of diabetes development by decreasing proinflammatory markers and maintaining intestinal barrier integrity, thereby assuming a protective role [18]. Consistent with these findings, the current study showed that the abundances of the *Lactobacillus* genus, which are related to probiotic functioning, decreased in the gut of female patients with DMC. However, to evaluate if this was associated with a reduction in the inflammatory response, further studies are required.

*Enterococcus* spp is a common urinary infection pathogenic bacterium, and the urethral perineum is susceptible to fecal flora pollution due to the specificity of the female body anatomy [19]. This study showed that the abundances of the *Enterococcus* spp in the T2DM-MC group were significantly higher than that in the T2DM-0 group. It is well documented that diabetes and its complications are characterized by systemic inflammation. As such, decreased abundances of anti-inflammatory bacteria, along with the increased abundances of proinflammatory bacteria is considered responsible for the occurrence and development of diabetic complications [20]. This could be one of the reasons for the increased abundances of *Enterococcus* spp in the T2DM-MC group. In addition, McMillan et al. [21] showed that *Lactobacillus* can destroy vaginal pathogen biofilms and inhibit the growth of vaginal pathogens in vitro, suggested that *Enterococcus* spp may overproliferate in T2DM-MC group without bioantagonism of *Lactobacillus*.

Lipopolysaccharide (LPS) is a critical component of the cell membrane of *Bacteroides* gram-negative bacteria. Previous study demonstrated that monocytes and macrophages were stimulated to release high mobility group B1 (HMGB1) protein, which is associated with increased LPS aggregation and infiltration into blood circulation [22]. HMGB1 binds to glycation end product receptors by activating the extracellular regulated protein kinase 1/2 signaling pathway and nuclear factor-*κ*B signaling pathway, aggravating microvascular inflammation and oxidative stress, resulting in retinal dysfunction and neurodegenerative changes [23]. This study showed that the abundances of *Bacteroides* genus were significantly higher in the females in the T2DM-MC group than in those in the T2DM-0 group. Whether the development of DMC in females occurs via the above mechanisms needs to be thoroughly evaluated.

The intestinal microbiome modulates the occurrence of DMC via various pathways, such as regulating glucose homeostasis and insulin resistance in metabolic organs, as well as microbiota metabolites interfering in glucose utilization. This study showed that HbA1c levels were significantly higher in the T2DM-MC group than in the T2DM-0 group, indicating that the blood glucose control was worse in patients with DMC. *Lactobacillus* was inversely correlated with HbA1c levels and 2hPG levels. Dang et al. [24] found that *Lactobacillus* possesses glucosidase-inhibitory activity and effectively reduces postprandial hyperglycemia by preventing carbohydrate decomposition. These findings are consistent with the results of the present study, which found that butyric acid-producing *Clostridium leptum* and *Roseburia inulinivorans* were negatively associated with HbA1c and FBG levels, respectively. Butyrate serves as a ligand for intestinal G protein-coupled receptor 41/43, promotes the release of intestinal hormones GLP-1, PYY, and GLP-2 in intestinal L cells, and plays an important role in blood glucose control [25]. This study also found that succinate-producing *Prevotella* bacteria was negatively associated with HbA1c levels, possibly related to reduced blood glucose levels through stimulation of intestinal gluconeogenesis via the inhibition of hepatic glucose output [26].

There are about 500-1000 species of gut microorganisms in humans and easy to be affected by multiple factors. This study excluded the interference of confounding factors, such as dyslipidemia, hypertension, large vascular complications, and diabetic drugs in the two groups. Additionally, *Bacteroides* have been found to be closely related to DMC in women, which pave the way for a new treatment strategy to regulate the balance of intestinal flora through the exogenous supplementation of probiotics. This can be used as an early intervention of the disease and can actively improve prognosis in the future.

There were some limitations in this study. First, our sample size is relatively small. All the participants are from the same region, and the serum samples can only represent the northern Chinese population. Second, when comparing T2DM-0 with T2DM-MC, there was a difference in age between the study population—T2DM-MC patients were older. These factors may have some effects on the differences between groups. Third, the mechanism about gut microbiota related to diabetic microvascular complications in female patients needs to be explored in further studies.

## 6. Conclusions

In the present study, gut microbiota is related to blood glucose levels. Female patients with DMC experience gut microbiota disorders. Our results demonstrate that there was a difference in the gut microbiota structure between the T2DM-0 and T2DM-MC groups. And the abundances of health-promoting bacteria (*Lactobacillus*) are lower than that in the T2DM-MC group. Taken together, our study provides new insights into the relationship between the gut microbiota and DMC in female patients. The current knowledge and studies can provide a new therapeutic strategy in battling T2DM and its complications. The regulation of gut microbiota through prebiotics, probiotics, synbiotics, or fecal microbiota transfer may have beneficial effects on the management of diabetes and associated complications.

## Figures and Tables

**Table 1:** Primer sequences of the intestinal bacterial genus.

Name	Upstream primer (forward 5′⟶3′)	Downstream primer (reverse 5′⟶3′)
*Bacteroides*	CTGAACCAGCCAAGTAGCG	CCGCAAACTTTCACAACTGACTTA
*Prevotella*	CCAGCCAAGTAGCGTGCA	TGGACCTTCCGTATTACCGC
*Bifidobacterium* spp	TCGCGTC (C/T)GGTGTGAAAG	CCACATCCAGC (A/G)TCCAC
*Lactobacillus*	AGCAGTAGGGAATCTTCCA	CACCGCTACACATGGAG
*Faecalibacterium prausnitzii*	CCCTTCAGTGCCGCAGT	GTCGCAGGATGTCAAGAC
*Enterococcus* spp	CCCTTATTGTTAGTTGCCATC	ACTCGTTGTACTTCCCATTGTATT
*Eubacterium rectale*	GGAATATTGCACAATGGGC	AGCCGGTGCTTCTTAGTCAG
*Veillonellaceae*	CCCGGGCCTTGTACACACCG	CCCACCGGCTTTGGGCACTT
*Clostridium leptum*	GCACAAGCAGTGGAGT	CTTCCTCCGTTTTGTCAA
*Roseburia inulinivorans*	TCTGACCGGACAGTAATGTG	CGCTGGCTACTGGGGATAAG
Internal parameters	CGTCAGCTCGTGYCGTGAG	CGTCRTCCCCRCCTTCC

**Table 2:** Clinical characteristics of the patients.

Variable	T2DM-0 group (**n** = 20)	T2DM-MC group (**n** = 17)	**t**/**X**2/**Z**	**P**
Age (years)	57.20 ± 7.44	62.00 ± 6.91	2.02	0.051
BMI (kg/m^2^)	24.30 ± 3.97	25.70 ± 2.91	1.203	0.237
Family history (%)	10 (50%)	7 (41.18%)	0.288	0.591
HbA1c (%)	8.48 ± 1.25	9.34 ± 1.26	2.091	0.044^∗^
FBG (mmol/L)	7.23 ± 1.88	8.35 ± 1.51	1.788	0.082
2hPG (mmol/L)	12.49 ± 2.98	13.92 ± 2.78	1.007	0.321
Hypertension (%)	12 (60.00%)	11 (64.71%)	0.087	0.769
Hyperlipidemia (%)	13 (65.00%)	12 (70.59%)	0.266	0.606
CRE (*μ*mol/L)	50.05 (43.00, 57.08)	60.4 (47.42, 85.69)	-1.417	0.156
Diabetic macrovascular complications	1 (5.00%)	2 (10.00%)	0.564	0.452
Medication status of antidiabetic agents				
Metformin (%)	18 (90.00%)	14 (82.35%)	0.46	0.498
*α*-Glycosidase inhibitor (%)	5	4	0.011	0.917
Insulin (%)	13 (65.00%)	13 (76.47%)	0.579	0.447

Abbreviations: T2DM-0: no diabetic microvascular complications; T2DM-MC: diabetic microvascular complications; BMI: body mass index; HbA1c: glycated hemoglobin A1c; FBG: fasting blood glucose; 2hPG: 2 h plasma glucose; CRE: serum creatinine. ^∗^*p* < 0.05 was considered statistically significant.

**Table 3:** Comparison of microbiota genus between the two groups.

Genus (%)	T2DM-0 group (*n* = 20)	T2DM-MC group (*n* = 17)	*Z*	*p* value
*Bacteroides*	16.09 (7.47, 33.04)	35.35 (17.55, 48.88)	-2.088	0.037^∗^
*Prevotella*	1.77 (0.22, 5.91)	0.71 (0.28, 2.46)	-1.189	0.235
*Bifidobacterium* spp	3.41 (1.03, 5.33)	4.52 (1.52, 9.06)	-1.128	0.259
*Lactobacillus*	1.11 (0.37, 3.03)	0.39 (0.15, 0.95)	-2.195	0.028^∗^
*Faecalibacterium prausnitzii*	6.53 (1.92, 11.23)	4.37 (1.98, 5.90)	-1.463	0.143
*Enterococcus* spp	0.05 (0.00, 0.37)	0.32 (0.08, 0.93)	-2.061	0.039^∗^
*Eubacterium rectale*	5.58 (1.48, 14.86)	4.04 (2.20, 10.32)	-0.213	0.831
Veillonellaceae	0.05 (0.01, 0.15)	0.05 (0.02, 0.13)	-0.429	0.668
*Clostridium leptum*	18.29 (5.49, 31.73)	23.21 (6.62, 31.03)	-0.427	0.67
*Roseburia inulinivorans*	0.23 (0.05, 0.60)	0.17 (0.06, 1.91)	-0.381	0.703

Abbreviations: T2DM-0: no diabetic microvascular complications, T2DM-MC: diabetic microvascular complications. ^∗^*p* < 0.05 was considered statistically significant.

**Table 4:** Logistic regression analysis of the effect of the *Bacteroides* genus on DMC.

Model	B	SE	Waldx2	*p* value	OR (95% CI)
1	0.044	0.022	4.196	0.041	1.045 (1.002–1.090)
2	0.038	0.038	4.716	0.03	1.087 (1.008–1.172)
3	0.059	0.059	4.29	0.038	1.130 (1.007–1.269)

Abbreviations: B: regression coefficient; SE: standard error of mean. OR: odd ratio; Model 1: logistic regression model, with *Bacteroides* as an independent variable; Model 2: adjusted for age, BMI, family history, and HbA1c levels; Model 3: model 2 additionally adjusted for hypertension, dyslipidemia, and creatinine levels. *p* < 0.05 was considered statistically significant.

## Data Availability

The data used to support the findings of this study are available from the corresponding author upon request.
